# “Holding on to Hope”: follow up qualitative findings of a tobacco treatment intervention for people experiencing mental health conditions

**DOI:** 10.3389/fpsyt.2024.1257112

**Published:** 2025-01-30

**Authors:** Tessa-May Zirnsak, Kristen McCarter, Melissa L. McKinlay, Ashleigh Guillaumier, Nadine Cocks, Catherine Brasier, Laura Hayes, Amanda L. Baker, Donita E. Baird, Billie Bonevski, Ron Borland, David Castle, Erin Forbes, Peter J. Kelly, Catherine Segan, Rohan Sweeney, Alyna Turner, Jill M. Williams, Lisa Brophy

**Affiliations:** ^1^ Social Work and Social Policy, School of Allied Health, Human Services and Sport, La Trobe University Melbourne, Melbourne, VIC, Australia; ^2^ School of Psychological Sciences, College of Engineering, Science and Environment, University of Newcastle, Callaghan, NSW, Australia; ^3^ Department of Mental Health, St Vincent’s Hospital Melbourne, Fitzroy, VIC, Australia; ^4^ Research, Advocacy and Policy Development, Mind Australia Limited, Heidelberg, VIC, Australia; ^5^ School of Medicine and Public Health, College of Health, Medicine and Wellbeing, University of Newcastle, Callaghan, NSW, Australia; ^6^ Flinders Health and Medical Research Institute (FHMRI), College of Medicine and Public Health, Flinders University, Bedford Park, SA, Australia; ^7^ Melbourne School of Psychological Sciences, The University of Melbourne, Melbourne, VIC, Australia; ^8^ Tobacco Control Unit, Cancer Council Victoria, Melbourne, VIC, Australia; ^9^ Department of Psychiatry, University of Tasmania; and Centre for Mental Health Service Innovation, Hobart, TAS, Australia; ^10^ School of Psychology, University of Wollongong, Wollongong, NSW, Australia; ^11^ Centre for Health Policy, Melbourne School of Population and Global Health, The University of Melbourne, Melbourne, VIC, Australia; ^12^ Centre for Health Economics, Monash Business School, Monash University, Melbourne, VIC, Australia; ^13^ School of Medicine, IMPACT, Institute for Innovation in Physical and Mental Health and Clinical Translation, Deakin University, Geelong, VIC, Australia; ^14^ Division of Addiction Psychiatry, Rutgers Robert Wood Johnson Medical School, New Brunswick, NJ, United States; ^15^ Centre for Mental Health, Melbourne School of Population and Global Health, The University of Melbourne, Melbourne, VIC, Australia

**Keywords:** tobacco treatment, quitline, peer worker, mental illness, severe mental illness, smoking cessation

## Abstract

**Background:**

Mental health service users are more likely to smoke tobacco and are as likely to make quit attempts as people not experiencing SMI, but they are less likely to succeed. Quitting tobacco can be harder for people experiencing SMI due to higher levels of nicotine dependence, more severe withdrawal, and many other complex factors. The Quitlink study was a randomized controlled trial combining a tailored 8-week Quitline intervention delivered by dedicated Quitline counsellors plus combination nicotine replacement therapy for people who experience SMI. The purpose of this paper is to report on the medium- and longer-term findings from interviews conducted at 5 and 8 months.

**Methods:**

As a part of the broader Quitlink study, participants were invited to qualitative interviews at 2, 5 and 8 months following recruitment, in line with quantitative follow-up time points. Interviews were conducted with 28 participants in the Quitlink trial (intervention group n = 12, control group n = 16). Interviews were transcribed and analyzed with a thematic analysis methodology using NVivo 12. Key themes were determined using inductive coding.

**Results:**

Six key themes were identified. These included: internal/external attributions for tobacco smoking, social relationships and relapse, the role of hopefulness in quitting, the role of clinicians in initiating and maintaining a quit attempt, increasing cessation literacy, and efficacy of the study intervention. Overall, findings suggested that participants’ quit attempts were often precarious and vulnerable, but active support and feelings of social connectedness were key to supporting participants to initiate a quit attempt and maintain gains.

**Conclusions:**

People who experience SMI can make attempts to quit smoking tobacco with support from clinicians and social networks. Connectedness and hope are significant enablers of making and sustaining quit attempts.

**Trial registration:**

The Quitlink trial was registered with ANZCTR (www.anzctr.org.au): ACTRN12619000244101 prior to the accrual of the first participant and updated regularly as per registry guidelines.

## Background

People who experience severe mental illness (SMI) are more likely to smoke tobacco and more likely to die from smoking-related causes than the general population (1–4). However, they are just as likely to have made or planned to make a quit attempt – defined by Chaiton et al. (5) as a period of abstinence with the intention of not smoking again – as people not living with SMI (6). Yet, cessation attempts for this group of people are less likely to be successful (7).

Challenges can include higher physical and psychological dependence on nicotine (8), more severe nicotine withdrawal (9, 10), and lack of support from health and other service providers (11) that may be related to perceptions that quitting smoking interferes with recovery from mental illness (12). A common challenge for achieving tobacco smoking cessation is that smoking can fulfill needs that are not being addressed elsewhere. For people living with SMI these needs may include alleviating isolation and despair associated with social exclusion and trauma (13).

Treatments that work in the general population (e.g. nicotine replacement therapy, focused psychological strategies) work for those with SMI and appear approximately equally effective (14). Best practice treatment for smoking cessation for people with or without mental illness combines multi-session behavioral intervention with pharmacotherapy (e.g., combination nicotine replacement therapy and/or a smoking cessation medication) (15). However, despite evidence-based interventions for tobacco treatment, the unique barriers experienced by people who experience SMI have meant gaining support and achieving success to quit has been challenging.

This qualitative study was a nested component of the ‘Quitlink’ study, a randomized controlled trial (RCT) of peer worker facilitated telephone support for smokers receiving mental health services. This was a tailored intervention for smokers who experience SMI, which has been described elsewhere (16–20).

Briefly, this study involved two conditions. The control condition included brief advice to quit from the peer worker, encouragement to use cNRT (combination nicotine replacement therapy) and to contact Quitline (a telephone support service), and provision of a pack of written materials which featured information about the Quitline service and motivational content relating to starting a quit attempt. Participants could call Quitline at their own discretion during the intervention period (2 months), and so the number and length of the calls varied (20). During the intervention period the average number of sessions were 3.3 (1-4) for the control and 5.2 for intervention group (1-15). The intervention condition included the same provision as the control condition, with the addition of a proactive referral to Quitline counselling based on cognitive behavioral therapy and tailored to meet the needs of people experiencing SMI, and up to 8 weeks of cNRT. Tailored Quitline counselling with a dedicated counsellor included structured monitoring of mental health symptoms, nicotine withdrawal symptoms, and medication side-effects to help distinguish temporary withdrawal symptoms from psychiatric symptoms; and a focus on psychoeducation including the relationship between smoking and mood; goal setting; identification of triggers to smoke; and facilitating problem solving and skills building, including the use of mood management strategies that also act to aid cessation (20).

Participants who completed qualitative interviews at the Quitlink 2 month follow-up timepoint identified enormous challenges when quitting (19). However, these interviews indicated that a tailored intervention had the potential to assist people on their journey to quitting while they continued their journey of mental health recovery (19). Through interviewing participants at medium and longer term timepoints (5 and 8 months), we sought to identify factors that sustained their attempts to quit and those that represented their most difficult challenges, thus complementing and building on the shorter term follow up findings. The purpose of this paper is to report on the medium- and longer-term findings from these interviews.

## Methods

Participants were invited to the Quitlink trial via face-to-face peer researcher facilitated recruitment. There were no formal relationships between participants and the Quitlink trial researchers prior to their recruitment in the main trial, although individual researchers were able to use contacts in the two partner organizations to facilitate recruitment. Ethical approval was granted by St Vincent’s Hospital, Melbourne (HREC Reference Number: HREC/18/SVHM/154), the University of Newcastle HREC (HREC Reference Number: H-2018-0192) and the Cancer Council Victoria, HREC (HREC Reference Number: 1807).

### Participants

In this qualitative sub-study, participants were those who participated in the ‘Quitlink’ RCT and provided consent to be contacted about participating in qualitative interviews at 2, 5 and 8 months post-recruitment. This paper reports findings from all participants in the Quitlink RCT who consented to be interviewed at the 5 or 8 month timepoints. Supply of pharmacotherapy ended at 2 months, but, as Quitline is a public service, participants could continue to access it after the intervention had ended. This paper is from participant’s reflections 3 and 6 months following the conclusion of the 2 month intervention period.

To be eligible for the main RCT, participants smoked at least 10 cigarettes a day and were accessing the two mental health services that were our organizational partners. When recruitment proved to be slower than expected it was supplemented by direct mail postcards and online recruitment methods. At this stage, inclusion criteria were also expanded to people who were accessing support or treatment (including from general practitioners) for mental health and/or alcohol or other drug problems beyond our initial two partner organizations to anywhere in the state of Victoria, Australia (18).

Delayed recruitment into the main trial and the impact of COVID-19 pandemic restrictions led to delays in commencing the qualitative interviews. Consequently, the first 25 people who had agreed to be contacted about an interview were already at the 5 or 8 month follow-up stage and missed the opportunity to be interviewed at the 2 month follow up.

Recruitment included the whole cohort of Quitlink participants and was not based on which condition (intervention or control) participants were placed in for the Quitlink trial. Participants were those who agreed to qualitative interviews. However, this meant that participants differed in how much they were exposed to the intervention. Participants were asked the same questions and overall, the identified themes reported in this paper are relevant to both the intervention and control groups. Our intention was not to make comparisons but some differences in experience between the control or intervention group participants did emerge, such as appreciating the continuity of care offered to those in the intervention.

### Procedures

Recruitment and interviewing were undertaken by a team of researchers including the peer researchers (NC, MM and CB) and the trial coordinator (KM).

Recruitment was conducted as a convenience sample with all Quitlink participants who provided consent to be contacted considered eligible to participate in the qualitative interviews. To facilitate engagement at the 5 or 8 month timepoints, researchers made telephone calls to people who had been recruited to the study to invite them to undertake a qualitative interview, including those who had already had a two month interview. Only one participant agreed to take part at both 5 and 8 months. As a result, there were 28 participants in the 5 and 8 months interviews but 29 total interviews.

Interviews were conducted via telephone with only the interviewer and interviewee present. Interviews were not conducted by the same person who recruited the participant to the main trial to reduce any potential for bias. A semi-structured interview guide was used (available as a Supplementary File). This guide was designed by the research team and modified based on specific advice from peer researchers. The interview guide included questions such as “what has been your experience of attempts to Quit?”, “Have you had different needs over time depending on whether you have attempted to quit or not, or relapsed?”, and “What role did any telephone counselling you have had play in your attempts to Quit?”.

The interviews were audio recorded and transcribed verbatim by a professional transcription service. Interviews varied in length, from 15 minutes to one hour. Participants received an AU$40 gift card per interview as remuneration for participation, consistent with the values outlined by the National Health and Medical Research Council (21). Following analysis of the data, participants were provided with an overall summary of findings, including key themes, and asked to contact the research team via post or reply email if they had any feedback.

### Data analysis

Thematic analysis, using a general inductive approach, was applied following Thomas (22), with no preconceived theories applied to the data. Thomas (22, 237) described the aims of inductive data analysis to be: ‘(a) condense raw textual data into a brief, summary format; (b) establish clear links between the evaluation or research objectives and the summary findings derived from the raw data; and (c) develop a framework of the underlying structure of experiences or processes that are evident in the raw data’.

TZ was recruited to the project after data collection had been completed. TZ and MM led the data analysis for this paper in collaboration with the full qualitative research team who met every two weeks to discuss the findings and agree on the coding and themes. Specifically, TZ conducted open coding of all transcripts independently of the other authors, using NVivo 12. MM contributed by creating their own codes and guiding a comparison with TZ’s codes. LB participated in the coding process by assisting TZ and MM with reviewing and refining existing codes. Axial coding was then performed by TZ to draw connections between codes. In collaboration with the rest of the team, the themes were identified and refined via consensus. As multiple lived experience researchers were employed on this project, there was opportunity to discuss and explore the findings of the research from multiple lived experience lenses.

Once the key themes were identified, the researchers posted out a one-page summary to all people who participated at 5 and/or 8 months. Participants had a period of just over one month to contact the research team to provide feedback, but none chose to do so.

### Patient and public involvement

Consumer engagement and accurate reporting of their contributions to research is an increasingly appreciated imperative in academic research (23). Our efforts to do this are reflected in the project by engaging peer researchers at all stages of the project. Several investigators (CB, NC, MM, TZ) were employed in consumer (or lived experience) roles and two had personal experiences of quitting tobacco smoking.

## Results

### Participants

**Table 1** indicates that the sample of 28 participants had slightly more men than women, most were single, and almost half were unemployed. Hence, participants in our study shared the demographic characteristics of many people who experience SMI (24), and were generally consistent with the characteristics of participants in the broader Quitlink RCT (20).

**Table 1:** Participant demographics.

Demographic	Total	Intervention	Control
	28	12	16
Gender (%)			
Male	16 (57.1)	6 (50.0)	10 (62.5)
Female	12 (42.9)	6 (50.0)	6 (37.5)
Mean Age (SD)	45.13 (10.51)	49.70 (9.019)	41.86 (10.560)
English as primary language (%)	26 (92.9)	12 (100.0)	14 (87.5)
Aboriginal or Torres Strait Islander Descent (%)	1 (3.6)	1 (8.3)	0 (0.0)
Marital status (%)			
Single, never married	14 (50.0)	3 (25.0)	11 (68.8)
Separated/Divorced	7 (25.0)	5 (41.7)	2 (12.5)
Married/De Facto	4 (14.3)	1 (8.3)	3 (18.8)
Widowed	2 (7.1)	2 (16.7)	0 (0.0)
Other	1 (3.6)	1 (8.3)	0 (0.0)
Has children under 18 (%)	5 (17.9)	3 (25.0)	2 (12.5)
Highest level of education (%)			
TAFE certificate, diploma, trade certificate or apprenticeship	13 (46.4)	5 (41.7)	8 (50.0)
School Certificate/Intermediate/Year 10/4^th^ Form	8 (28.6)	2 (16.7)	6 (37.5)
University/College of Advanced Education/some other tertiary institute degree or higher	3 (10.7)	1 (8.3)	2 (12.5)
Years 7 to 9	2 (7.1)	2 (16.7)	0 (0.0)
HSC/Leaving/Year 12/6^th^ Form	1 (3.6)	1 (8.3)	0 (0.0)
Other	1 (3.6)	1 (8.3)	0 (0.0)
Current employment (%)			
Unemployed	13 (46.4)	4 (33.3)	9 (56.3)
Part time or casually employed	6 (21.4)	2 (16.7)	4 (25.0)
Employed full time	3 (10.7)	2 (16.7)	1 (6.3)
Studying	3 (10.7)	1 (8.3)	2 (12.5)
Other	2 (7.1)	2 (16.7)	0 (0.0)
Stay at home parent	1 (3.6)	1 (8.3)	0 (0.0)

Most participants were recruited from the study’s two partner organizations: a large inner city clinical mental health service, and a state-wide community mental health support service (a non-government agency). Both offered a range of residential and community-based services to people living with mental health conditions (**Table 2**). **Table 3** below outlines the number of participants who completed 5 or 8 month interviews, whether they were in the control or intervention group, and any change in smoking status.

**Table 2:** Recruitment Pathways.

	Residential (%)	Community (%)	Online (%)
Intervention	0 (0.0)	10 (35.7)	2 (7.1)
Control	1 (3.6)	13 (46.4)	2 (7.1)
Total	1 (3.6)	23 (82.1)	4 (14.3)

**Table 3:** Changes in smoking behavior of participants.

	Number of participants	Timepoint of final interview		Change in smoking behavior at the time of final interview (5 or 8 months)*			
		5 months	8 months	No change	Cut down	Quit or cut down and relapsed^𐠒^	Quit (no relapse)
Intervention	12	4	8	1	4	3	4
Control	16	9	8	4	7	4	1
Total	28	12	16	5	11	7	5

*only one participant completed an interview at 5 and 8 months. Change in smoking behavior for this participant is reported at 8 months.

^𐠒^Defined as 7+ days of continuous smoking, with no reported smoking in the previous week (16),

Of the 13 participants interviewed at 5 months, four were also interviewed at 2 months. Of these participants, two quit, one cut down, and one relapsed. Of the 16 participants interviewed at 8 months, 10 were also interviewed at 2 months. Of these participants, six cut down, two relapsed and two quit. One participant was interviewed at 5 and 8 months.

No participants completed interviews at all 2, 5, and 8 month time-points. Hence, there was limited opportunity to consider individual participants’ situations in a longitudinal way, and our analysis focused on specific points in time. Regardless, the data participants provided illuminated the challenges of smoking cessation and/or reduction and demonstrated how reflections on tobacco smoking by people living with mental illness may change over time.

### Themes

Six key themes were identified. These were: internal and external attributions for smoking, social relationships, the role of hopefulness, the role of clinicians, increasing cessation literacy, and engagement and connection during the study. In this section, we address each of these themes in turn. Each theme is outlined in detail below, and **Table 4** contains a brief outline of the key finding associated with it.

**Table 4:** Summary of identified themes.

Theme	Summary
Internal and external attributions for smoking	Participants felt that their quit attempts failed because of factors outside of them (e.g. COVID-19, relationship breakdown) or because of their emotional resources (e.g. willpower, resilience).
Social relationships	Interpersonal connections enabled and challenged participants. Relationships with supportive and non-smoking peers helped participants to maintain motivation, while spending time with peers who smoked created vulnerability to relapse.
The role of hopefulness	Participants found that believing that they could quit was essential to making or continuing a quit attempt.
The role of clinicians	Support from clinicians in making a quit attempt was essential, but participants did not find unsolicited advice to quit useful.
Increasing cessation literacy	Participants in both cohorts valued the support offered by the Quitlink study, but much of this was publicly available, suggesting that there is a need for increased communication regarding these resources to people living with SMI.
Engagement and connection with the broader Quitlink study	Participants found that the continuity offered by the Quitlink study was useful in their attempts to quit.

#### Internal/external attributions for smoking

*‘I’ve relapsed a couple of times the last few months – but that’s only because I have had difficult situations in my life’* – Participant 92, intervention, quit and relapsed.

As the above quote suggests, it appears that participants at 5 or 8 months post recruitment were becoming more aware of external and internal factors that influenced their cessation attempts. For the above participant, experiencing stressors was the catalyst for relapse externally generated (rather than psychological) barriers remained significant for some participants. For example, one participant referenced COVID-19 as a significant external factor that formed a barrier to quitting. They said:

*‘But I think that that was a really, really bad time to – I guess even be alive, yeah. [ … ] I guess my quit smoking was a failure’* – Participant 41, intervention, cut down and relapsed to previous levels.

However, some participants described the reason that their quit attempt had been unsuccessful as internal, highlighting their own psychological readiness as imperative to a successful quit attempt. One participant said:

*‘I did try, but no I’m not ready, I’m really not ready’* – Participant 25, control, cut down.

Another participant was more forthcoming about the impact of psychological readiness on their ability to cut down or make a quit attempt:

*‘It’s a matter of discipline if you’re disciplined enough in what you set your mind to, you’re more likely to achieve what you set out for yourself and discipline is what I lack’* – Participant 49, control, cut down.

These experiences indicate that internal psychosocial resources were commonly indicated as imperative to quitting smoking by participants. For this reason, advice to quit is not sufficient to facilitate a quit attempt, it requires a commitment from the person. One participant articulated this need, claiming:

*‘But you’ve got to be, your mind’s got to be in it, you know what I mean, it’s not enough for someone to say just quit today, you’ve got to be ready to quit’* – Participant 30, intervention, no change.

#### Social relationships and relapse

*‘If I’d see [my friend] [ … ] if he’s got a smoke, I’d just grab it and have a couple of drags and give it back to him. [ … ] Once you’d had that little drag, that was it’* – Participant 34, intervention, cut down.

Social relationships influence could be both helpful and detrimental in maintaining a quit attempt. As the participant above outlines, circumstantial contact with a friend who smokes was likely to lead to relapse and one “drag” was all the was required to end their quit attempt.

Another participant described having the support of other people who had also quit smoking helped them to see quitting as possible for themselves. They said:

*‘My other brother has managed to quit, and he only smokes a vape, so his example is a good support’* – Participant 74, intervention, cut down.

Another participant highlighted that this positive influence could also be found in non-smoking intimate partners. While this participant had not experienced a current change in their smoking, they reflected on a time in their life when they had been able to quit with the support of a partner:

*‘When I quit in my 20s my husband helped me through. And that made a big difference, I think’* – Participant 30, control, no change.

These experiences indicate that our participant’s quit attempts were vulnerable to relapse – but that active support from others was a protective factor. One participant presented an idea for how practical support from their daughter could be helpful in maintaining their quit attempt:

*‘If someone had control of my money, which I’ve actually spoken to my younger daughter about, [ … ] at least then I can’t go and buy tobacco for 10 bucks. Just I think she’d be strong enough to stand her ground and say “no Mum”‘* – Participant 34, intervention, cut down.

Thus, participants experienced their social relationships as potential enablers of cessation and triggers for relapse based on if the person smoked, and if they were supportive of the quit attempt.

#### The role of hopefulness in quitting

Participants found that believing that a future where they didn’t smoke was possible facilitated making and maintaining a quit attempt. Conversely, participants who did not feel that a different future was possible described a lack of hopefulness as a barrier to quitting. One participant said:

*‘I find at night I am very sad at night and depressed [ … ] some nights I feel like I should go out and get a packet of smokes just to sort of you know [ … ] I just find at night that I find it difficult’*– Participant 92, intervention, quit.

For this participant, being able to smoke helped them with their feelings of sadness, suggesting that smoking was of some comfort to them. Without being able to smoke, participants may need other ways to cope with negative emotions that have previously been addressed by smoking.

Another participant expressed that their capacity to see a different future for themselves was hindered by the COVID-19 pandemic:

*‘I could save money and I could go on holidays, or I could buy this, but you can’t plan anymore for anything you can’t, there’s just no reason you know there’s no reason, no future, no hope’* – Participant 54, control, no change.

The feelings of hopelessness and despair that can accompany living with SMI was a barrier to being able to believe a quit attempt would be successful. The comments from these participants suggest that hope is essential in deciding to make a quit attempt –participants were less likely to make a quit attempt if they did not have hope they could quit. Lack of hope impacted other aspects of participant’s lives. One participant said:

*‘When I’m not feeling very hopeful it’s pretty hard to sort of motivate yourself with something’* – Participant 52, intervention, cut down.

Participants who did not have hope that they could stop smoking lost motivation to continue their quit attempt, as they saw little value in suffering for a better future:

*‘I’m really hoping, I don’t want to continue smoking but I just can’t see my way forward [ … ]I made a promise to a grandchild and I broke that promise you know, when he was quite young he said to me can you give up Nan, and I went yeah just for you I’m going to do it, and I broke that promise’* – Participant 25, control, no change.

Holding hope that a successful quit attempt was possible seemed to serve the participants in our study. Participants did experience hope about their potential to quit through access to the support provided both for the control and intervention conditions (noting that the intervention cohort received more proactive support). One said that the Quitline staff:

*‘Told me a lot that I could do it even though I do suffer from mental illness and stress and … that it was still possible’* – Participant 95, intervention, no change.

#### Role of clinicians in initiating and maintaining a quit attempt

All participants in the current interviews reported thinking about or making quit attempts. Having clinicians supporting quit attempts was important to participants. For one participant, they reminded them to quit smoking and cultivated a sense that the person was not alone in their quitting journey. They said:

*‘The fact that all the health professionals involved in my mental health were pushing for me to quit was good for my mental health as well, so I had them supporting me’* – Participant 49, control, cut down.

However, active support was not the norm. One participant shared that their General Practitioner (GP) had subordinated their smoking attempt to their mental health recovery, suggesting that clinicians are wary to encourage smoking cessation if they perceive this goal to conflict with the person’s mental wellbeing:

*‘He had no concerns about me smoking which I thought was unusual because normally they sort of discourage that sort of thing, [ … ] he knows what I’ve gone through for the last 18 months too so maybe he thought that [smoking] might help me mentally’* – Participant 92, intervention, quit and relapsed.

Another explained that the treatment offered by GPs is only useful in the context of the patient-clinician relationship. Clinicians simply suggesting standard treatments were not sufficient for participants to feel supported in their quit attempt. This participant said:

*‘GPs handing out NRT is such a blanket response you know – yeah – unless you’ve got a really good rapport, not a good rapport but if you’ve got rapport, he might kind of talk to you a bit about it or give you some information or flyers or websites to look up, but the one I’ve got is more yeah – not really that helpful’* – Participant 96, control, cut down and relapsed

Findings in this section suggest that giving people experiencing SMI the option of active support, without forcing it on them, was preferred.

#### Increasing cessation literacy

*‘I mean there’s a lot of people out there that really struggle with [ … ] their quit attempts, and then I found with the support they provided me with extra guidance, and so pointed me in the right direction along the way’* – Participant 31, intervention, quit.

Participants indicated that they had a desire for more information about smoking, quitting, and available services like appointments with clinicians, mental health support and cessation-specific support. This is linked to the last theme and indicates that participants did not want to make cessation attempts without support. One participant outlined how knowledge of services that came about through their participation in the study was essential to them being able to accept further help:

*‘Now I’m aware that they’re there and that I can use them for whatever I may need at that time, it’s I guess, it’s empowering, so to now know that they’re there and that you can utilize these things is good, it is really good’* – Participant 41, intervention, cut down.

However, one participant in the control group expressed that the information they needed to motivate them to quit was only provided to them through their participation in the study – even though the control condition were only provided with publicly available services. As such, one of their key motivations for enrolling in the study was to understand more about how their smoking impacts their body:

*‘there’s a lot of things that are wrong and bad for you, but I am more concerned about my body – and whether my body feels bad when I am smoking cigarettes [ … ] yeah, that’s what I am trying to learn – what does a cigarette come down to[]?’* – Participant 83, control, quit.

This theme and the supporting quotes suggest that information helped participants to feel empowered to quit and strengthened their resolve. That control participants may not have been able to access publicly available information outside the context of the trial suggests that participants benefitted from the connections with researchers as a part of their participation in the trial.

#### Engagement and connection in the course of the study

Participants in the intervention group found the Quitlink intervention elements (Quitline, cNRT and peer researcher engagement) useful to varying degrees, often depending on their circumstances and their existing relationships with clinicians. One said:

*‘It probably would’ve been better if the situation wasn’t what it was, but I felt like the support was probably the only thing that kept me in touch with the outside world through all this’* – Participant 41, intervention, cut down.

Participants in the intervention cohort found the continuity of having the same counsellor useful. They found that having someone available to connect with helped them to continue their quit attempt. One participant said:

*‘You know there’s always someone there, it may not be my counsellor, but no I would never have used the excuse of you know only being able to speak to my counsellor once a week [ … ] A lot of people probably would benefit from [ … ] their own personal counsellor’* – Participant 34, intervention, cut down.

Participant 34 is also highlighting the value of having their counsellor nested in a service that can provide support around the clock – even if their usual counsellor isn’t available.

Some control participants were reluctant to call Quitline for a variety of reasons. One said:

*‘I suppose I don’t really like counselling services ‘* – Participant 40, control, cut down.

Some participants indicated that there was a need to have more engagement from peer workers and peer researchers, rather than reliance on counselors who may not have experience of SMI or quitting themselves. When asked directly about the role of peer researchers in the intervention, one participant in the control group (who did not have regular calls from a Quitline counsellor) said:

*‘Having you guys more would be some form of motivation [ … ], if you have someone checking on your progress frequently, you’re less likely to fall back on to your bad habits – because it will take a couple of weeks and I’d forget about you guys and then back at it again or I increase or something’* – Participant 49, control, cut down.

Peer workers and Quitline counsellors also had a role in helping participants to feel connected even independently from their cessation attempt. One participant described how this contributed positively to their life:

*‘It was just nice to speak to somebody you know – on my isolated, I’m on my own, just nice to speak to somebody even if it was a few minutes – [ … ] nice to have that kind of connection’* – Participant 92, intervention, cut down.

## Discussion

The results from this study highlight the significance of hopefulness in making and maintaining a quit attempt. The findings suggest that people experiencing SMI who smoke may benefit from engagement with professionals who can hold on to the hope that they can quit over the long term. These findings can be used to inform further research into enablers of smoking cessation and guide clinicians in the support offered to people experiencing SMI who smoke.

The interviews at 2 months highlighted that people who experience SMI face enormous challenges when quitting (19). The findings at this timepoint suggested that a tailored smoking treatment intervention has the potential to assist people to quit, and that compassionate support encapsulating a recovery-oriented approach is highly valued. In the short term, participants were more likely to identify their smoking challenges as external to themselves – participants mentioned factors such as ‘grief, homelessness, trauma, their poor mental health and other problematic substance use’ as barriers to quitting (19, 5). Participants ‘described a lack of desire to quit smoking as being linked to living with their mental health challenges, including the will to live, and not feeling deserving of a better, healthy life’ (19, 7).

Findings in this paper corroborate the findings at the 2 month follow up timepoint of our study (19), but with some key differences. In the later follow up interviews, it appears that many participants had reframed what the barriers and enablers for smoking cessation were for them, with emphasis on new themes. This included greater awareness of how their willingness to quit served to increase the likelihood of their quit attempt being successful. Readiness to quit was initially identified as a theme at 2 months, and by 5 and 8 months, participants had been persistently invited to reflect on their smoking in follow up interviews, which enabled greater recognition of the patterns of behavior and thinking linked to their attempts to quit.

Participants in our study illustrated how an unsuccessful quit attempt could help them to recognize why they were smoking and address this in a future quit attempt. As such, many participants did not recognize an unsuccessful attempt to quit as a negative outcome in and of itself, but rather found the experience gained from repeated quit attempts to be opportunities for learning.

Barriers to quitting for participants included a lack of hope that a different (non-smoking) future is possible. Like Twyman et al. (25), the findings in this study indicate that a lack of hope or optimism on the part of clinicians can impact the quit attempts of service users. Low mood challenged the capacity of participants to see positive futures for themselves, encouraging pessimism and lack of confidence that the journey to quit smoking will be worth the effort – suggesting that lack of hopefulness may be a more significant barrier for people experiencing SMI. A systematic review by Zabeen et al. (26) on physical health support for people who experience SMI (which included some analysis of smoking cessation as a secondary aim) similarly illustrated that this group struggled to hold on to the hope that their physical health could be better. However, caregivers in their study were able to hold on to hope for people who experience SMI, which was associated with positive physical health outcomes. Mental distress remained an ongoing challenge that participants in our study were facing well beyond the conclusion of the intervention; this was also found in other, similar studies (27), reflecting an ongoing need for encouragement and support.

We found that that quitting is possible for people who experience SMI, but that their quit attempts were vulnerable to relapse. However, these attempts benefit from connections with others who believe that the person can quit. Clinicians working with participants in this study sometimes did not address smoking behavior in favor of addressing other mental or physical health concerns. While mixed support from clinicians has been identified in another study by Prochaska (12), neglecting the capacity for smoking cessation among people who experience SMI is a health justice issue (4). Neglect of smoking behavior on the part of clinicians may be related to the commonly held yet disproven myth that people who experience SMI are at an unacceptable risk of increased distress if they attempt to quit smoking (28). This is consistent with other examples of risk aversion being a prominent feature of mental health service delivery, linked to fear and sometimes stigma (4, 25, 29).

In a study by Hammett et al. (30), participants in the US with and without the experience of SMI were more likely to utilize cessation treatments if they were encouraged to do so by a health practitioner. This illustrates the important role that clinicians have in helping people to initiate a quit attempt. The same study found that ‘smokers with SMI utilized cessation treatments at higher rates than those without SMI’, although the authors indicate that this may be because people who experience SMI were more likely to have a regular health care provider who they were familiar with (30, 6).

The present study confirms there is a need for clinicians, such as GPs, case managers and other formal supporters to provide active support for people who experience SMI attempting to quit smoking. Clinicians should follow the evidence and not inappropriately wait for what they see as the best conditions for a quit attempt. This may result in lost opportunities to support a quit attempt. Clinicians can assist by confirming with patients that smoking treatment is likely to be of benefit to both their physical and mental health and serve as an anchoring point for smoking cessation encouragement and resources. It also appears that brief advice is insufficient to help people experiencing SMI to quit (31). As suggested by McCarter et al. (19), participants need to be supported as individuals with complex and diverse needs that do not necessarily mean that quitting is not possible.

Informal social networks also play an important role in supporting people during a relapse or following a quit attempt, and this was apparent in our study. Evidence from a trial of people who experience SMI in the United States found that social relationships were key to commencing a quit attempt and preventing or enabling relapse (32). As with our study, more participants in Aschbrenner et al.’s (32) study made and continued a quit attempt with support from their social network. However, many of the participants in both Aschbrenner et al.’s (32) study and the current study identified pro-smoking norms in their social circles, including amongst social connections they had made by smoking in mental health services, suggesting that social networks conducive to successful quit attempts may be difficult to cultivate.

Smoking may have a role in addressing people’s unmet needs, including social isolation and boredom (4). It appears that the Quitlink intervention was useful in helping people who experience SMI to feel connected. There is evidence that people who experience SMI also experience social isolation at higher rates than the general population (33). Including opportunities for connection – through support groups, peer mentoring, etc. – in smoking interventions may be a way to support people who experience SMI beyond the goal of smoking cessation.

The findings in this study suggest that people who experience SMI require consistent, non-judgmental support to quit smoking. Participants seemed to benefit from relationships where others believed they were capable of quitting smoking, or when they held this belief themselves. Findings from the 5 or 8 month interviews suggest that a compassionate, hopeful, and supportive approach must incorporate family, friends and other mental health clinicians and health service providers that inform the context in which the quit attempt is made. In this way, people who experience SMI making a quit attempt are centered in a whole of community approach.

Evidence based strategies, such as cNRT and Quitline counselling, were appreciated by participants in the long term, especially when these encouraged more self-efficacy and hope that quitting was possible. However, findings suggest the need for innovation in how smoking efforts can be sustained and relapses prevented. For example, support groups and 1:1 support matched based on participants values and needs warrant further investigation. Further efforts to involve family members, friends, peer workers and clinicians to support quit attempts and acknowledging that being in or creating relationships where quitting is encouraged and supported may be essential to successful smoking cessation by people who experience SMI.

### COVID-19

Our previous paper on recruitment for this study outlined that engagement with potential participants was hampered by the impact of COVID-19 (18). However, COVID-19 continued to have an impact on participants after they were recruited to the study.

Another similar study by Leutwyler and Hubbard (34) found that the COVID-19 pandemic and its associated disruption in routine was found to be a barrier to quitting tobacco smoking, as people experienced more stress and boredom – two factors associated with increased tobacco smoking.

In our study, some participants made direct reference to COVID-19, largely to indicate how health restrictions increased the social isolation that made their quitting journey more difficult.

### Limitations

One limitation of the study was the lack of longitudinal qualitative data on individual participants. Due to slow recruitment, participant availability, and complications due to the COVID-19 pandemic, only one participant did both 5 and 8 month interviews, limiting the capacity for a meaningful comparison between these timepoints. Further, this study had a relatively small sample size and is not intended to be generalizable. Regardless of these limitations, the research team were able to contribute to improved understanding of the experiences of the participants of the overall study at different time points post intervention. These insights will support the development and implementation of future smoking cessation interventions with people with SMI.

People with English as a first language were overrepresented in this study. Participants were mostly those recruited through community mental health services, and so people accessing residential services – and consequently those that may have higher needs than people living in the community – were not well represented in this study. The small numbers and difference in social demographic characteristics of participants from the control and intervention groups means that comparisons need to be interpreted with caution.

## Conclusion

It is possible for people who experience SMI to successfully quit smoking. Overall, the participants in our study demonstrated that every smoking attempt – no matter the outcome – has value on the journey to quitting. We believe that this is the primary strength of this study. We found that for many of our participants, a quit attempt can lead to improved awareness of factors important to their next attempt – including their social circumstances and psychological readiness. People who live with the impact of SMI need others in their formal and informal network of supporters to be encouraging and ‘hold the hope’, especially when a quit attempt is unsuccessful. People living with SMI often have complex needs that must be addressed to support smoking cessation. Despite restrictions on access to cigarettes generated by public health measures, people who experience mental health conditions encounter frequent exposure to cigarettes and other people who smoke – making quitting a bigger challenge and this may be a factor that contributes to the gap between those with mental health conditions and the rest of the community regarding smoking rates. Future interventions should focus on how to address the social as well as physical and psychological needs of this group to help increase rates of sustained smoking cessation.

## Data Availability

The raw data supporting the conclusions of this article will be made available by the authors, without undue reservation.

## References

[1] Cocks N Brophy L Segan C Stratford A Jones S Castle D. Psychosocial factors affecting smoking cessation among people living with schizophrenia: A lived experience lens [Perspective. Front Psychiatry. (2019) 10:565. doi: 10.3389/fpsyt.2019.00565 31474884 PMC6704230

[2] Klein P Lawn S Tsourtos G Joep van A. Tailoring of a smartphone smoking cessation app (Kick.it) for serious mental illness populations: qualitative study. JMIR Hum Fact. (2019) 6:1–15. doi: 10.2196/14023 PMC6754228 31482850

[3] Hawes MR Roth KB Cabassa LJ. Systematic review of psychosocial smoking cessation interventions for people with serious mental illness. J Dual Diagn. (2021) 17:216–35. doi: 10.1080/15504263.2021.1944712 PMC8647929 34281493

[4] Jenkin G McIntosh J Hoek J Mala K Paap H Peterson D. There’s no smoke without fire: smoking in smoke-free acute mental health wards. PloS One. (2021) 16:e0259984. doi: 10.1371/journal.pone.0259984 34780542 PMC8592473

[5] Chaiton M Diemert L Cohen J Bondy S Selby P Philipner A. Estimating the number of quit attempts it takes to quit smoking successfully in a longitudinal cohort of smokers. BMJ Open. (2016) 6:1–9. doi: 10.1136/bmjopen-2016-011045 PMC4908897 27288378

[6] Kalkhoran S Thorndike AN Rigotti NA Fung V Baggett TP. Cigarette smoking and quitting-related factors among US adult health center patients with serious mental illness. J Gen Internal Med. (2019) 34:986–91. doi: 10.1007/s11606-019-04857-3 PMC6544702 30783880

[7] Greenhalgh EM Brennan E Segan C Scollo M. Monitoring changes in smoking and quitting behaviours among Australians with and without mental illness over 15 years. Aust New Z J Public Health. (2022) 46:223–9. doi: 10.1111/1753-6405.13185 34821438

[8] Williams JM Steinberg ML Griffiths KG Cooperman N. Smokers with behavioral health comorbidity should be designated a tobacco use disparity group. Am J Public Health. (2013) 103:1549–55. doi: 10.2105/AJPH.2013.301232 PMC3776478 23865661

[9] Smith PH Homish GG Giovino GA Kozlowski LT. Cigarette smoking and mental illness: A study of nicotine withdrawal. Am J Public Health. (2014) 104:e127–33. doi: 10.2105/AJPH.2013.301502 PMC3935702 24328637

[10] Reid HH Ledgerwood DM. Depressive symptoms affect changes in nicotine withdrawal and smoking urges throughout smoking cessation treatment: preliminary results. Addict Res Theory. (2016) 24:48–53. doi: 10.3109/16066359.2015.1060967 27547173 PMC4988686

[11] Twyman L Bonevski B Paul C Bryant J. Perceived barriers to smoking cessation in selected vulnerable groups: A systematic review of the qualitative and quantitative literature. BMJ Open. (2014) 4:e006414. doi: 10.1136/bmjopen-2014-006414 PMC4275698 25534212

[12] Prochaska JJ. Smoking and mental illness — Breaking the link. New Engl J Med. (2011) 365:196–8. doi: 10.1056/NEJMp1105248 PMC4457781 21774707

[13] Keller-Hamilton B Moe AM Breitborde NJK Lee A Ferketich AK. Reasons for smoking and barriers to cessation among adults with serious mental illness: A qualitative study. J Community Psychol. (2019) 47:1462–75. doi: 10.1002/jcop.22197 31102293

[14] Banham L Gilbody S. Smoking cessation in severe mental illness: what works? Addiction. (2010) 105:1176–89. doi: 10.1111/j.1360-0443.2010.02946.x 20491721

[15] United States Department of Health and Human Services. Smoking Cessation: A Report of the Surgeon General. US Department of Health and Human Services. Georgia, USA: National Center for Chronic Disease Prevention and Health Promotion (2020).

[16] Baker AL Borland R Bonevski B Segan C Turner A Brophy L. Quitlink”—A randomized controlled trial of peer worker facilitated quitline support for smokers receiving mental health services: study protocol. Front Psychiatry. (2019) 10:124. doi: 10.3389/fpsyt.2019.00124 30941063 PMC6434698

[17] Sweeney R Moodie M Baker AL Borland R Castle D Segan C. Protocol for an economic evaluation of the quitlink randomized controlled trial for accessible smoking cessation support for people with severe mental illness. Front Psychiatry. (2019) 1–15. doi: 10.3389/fpsyt.2019.00618 PMC6735263 31551827

[18] Baker AL McCarter K Brophy L Castle D Kelly PJ Cocks N. Adapting peer researcher facilitated strategies to recruit people receiving mental health services to a tobacco treatment trial. Front Psychiatry. (2022) 13:869169. doi: 10.3389/fpsyt.2022.869169 35722563 PMC9199858

[19] McCarter K McKinlay ML Cocks N Brasier C Hayes L Baker AL. The value of compassionate support to address smoking: A qualitative study with people who experience severe mental illness. Front Psychiatry. (2022) 13:868032. doi: 10.3389/fpsyt.2022.868032 36276321 PMC9583161

[20] Baker AL McCarter K Turner A Segan C Castle D Brophy L. [amp]]lsquo;Quitlink’: Outcomes of a randomised controlled trial of peer researcher facilitated referral to a tailored quitline tobacco treatment for people receiving mental health services. Aust New Z J Psychiatry. (2023) 58(3): 260–276. doi: 10.1177/00048674231181039 PMC10903138 37353970

[21] National Health and Medical Research Council. National Statement on Ethical Conduct in Human Research. Canberra, Australia: National Health and Medical Research Council (2018). Available at: https://www.nhmrc.gov.au/about-us/publications/national-statement-ethical-conduct-human-research-2007-updated-2018.

[22] Thomas DR. A general inductive approach for analyzing qualitative evaluation data. Am J Eval. (2006) 27:237–46. doi: 10.1177/1098214005283748

[23] Gray R Brasier C Zirnsak T-M Ng AH. Reporting of patient and public involvement and engagement (PPIE) in clinical trials published in nursing science journals: A descriptive study. Res Involve Engage. (2021) 7:88. doi: 10.1186/s40900-021-00331-9 PMC8669663 34906260

[24] Morgan VA Waterreus A Jablensky A Mackinnon A McGrath JJ Carr V. People living with psychotic illness in 2010: the second Australian national survey of psychosis. Aust New Z J Psychiatry. (2012) 46:735–52. doi: 10.1177/0004867412449877 22696547

[25] Twyman L Cowles C Walsberger SC Baker AL Bonevski B Tackling Tobacco Mental Health Advisory Group. [amp]]lsquo;They’re going to smoke anyway’: A qualitative study of community mental health staff and consumer perspectives on the role of social and living environments in tobacco use and cessation. Front Psychiatry. (2019) 10:503. doi: 10.3389/fpsyt.2019.00503 31379622 PMC6652148

[26] Zabeen S Phua D Mohammadi L Lawn S. Family involvement to support cardiovascular self-management care for people with severe mental illness: A systematic review. J Ment Health. (2020), 1–17. doi: 10.1080/09638237.2020.1818194 32924668

[27] Lum A Skelton E Wynne O Bonevski B. A systematic review of psychosocial barriers and facilitators to smoking cessation in people living with schizophrenia. Frontier Psychiatry. (2018) 9:565. doi: 10.3389/fpsyt.2018.00565 PMC6232499 30459658

[28] Kertes J Stein Reisner O Grunhaus L Neumark Y. The impact of smoking cessation on hospitalization and psychiatric medication utilization among people with serious mental illness. Subst Use Misuse. (2021) 56:1543–50. doi: 10.1080/10826084.2021.1942057 34193007

[29] Smith CA McNeill A Kock L Ahmed Z Shahab L. Mental health professionals’ Perceptions, judgements and decision-making practices regarding the use of electronic cigarettes as a tobacco harm reduction intervention in mental healthcare: A qualitative focus group study. Addictive Behav Rep. (2019) 10:100–84. doi: 10.1016/j.abrep.2019.100184 PMC6545441 31193875

[30] Hammett PJ Taylor BC Lando HA Widome R Erickson DJ Fu SS. Serious mental illness and smoking cessation treatment utilization: the role of healthcare providers. J Behav Health Serv Res. (2021) 48:63–76. doi: 10.1007/s11414-020-09707-3 32378032 PMC7644603

[31] Steinberg ML Williams JM Stahl NF Budsock PD Cooperman NA. An adaptation of motivational interviewing increases quit attempts in smokers with serious mental illness. Nicotine Tobacco Res. (2015) 18:243–50. doi: 10.1093/ntr/ntv043 PMC4750086 25744954

[32] Aschbrenner K Patten CA Brunette MF. Feasibility of a support person intervention to promote smoking cessation treatment use among smokers with mental illness. Transl Behav Med. 8(5):785–92. doi: 10.1093/tbm/ibx033 PMC6128961 29385555

[33] Morgan VA Waterreus A Carr V Castle D Cohen M Harvey C. Responding to challenges for people with psychotic illness: updated evidence from the survey of high impact psychosis. Aust New Z J Psychiatry. (2016) 51:124–40. doi: 10.1177/0004867416679738 27913580

[34] Leutwyler H Hubbard E. Telephone based smoking cessation intervention for adults with serious mental illness during the COVID-19 pandemic. Tobacco Use Insights. (2021) 14:1–5. doi: 10.1177/1179173X211065989 PMC8721361 34987298

