# Supplementary material for: “Holding on to Hope”: follow up qualitative findings of a tobacco treatment intervention for people experiencing mental health conditions
Source: Front Psychiatry. 2025 Jan 30;15:1257112. doi: 10.3389/fpsyt.2024.1257112 (PMC11841444; doi:10.3389/fpsyt.2024.1257112)
Supplement: Supplementary file 1 [file DataSheet1.pdf]

Supplementary File: Interview Questions for "Holding on to Hope": Follow up qualitative findings of a tobacco treatment intervention for people experiencing mental health conditions

These will be semi structured interviews, so this is only a guide to the interviews. The interviewer may explore some responses in more depth.

At 5 months

- What has been your experience of attempts to Quit? Have you been successful? Have you relapsed? What do you think has been helpful or not helpful in your attempts to Quit? If you relapsed, why do you think this happened? What are the barriers you have faced in your attempts to quit (prompts – environment, support people, smoking culture, mental health issues)
- What has been your experience of getting help to Quit?
- Have you had assistance from peer workers? Do you think your attempts to quit were enhanced by involving peer workers? Why? How?
- Is there anything that could have been done differently to enhance the role of peer workers?
- What is your impression of the Quitline or other telephone counselling? (prompts – strengths, weaknesses, things you think could be done differently)
- What role did any telephone counselling you have had play in your attempts to Quit? Are there any other things that have been helpful (prompts – NRT, peer workers, engagement with mental health services and support network, peer worker follow up?). Please explain your thoughts on this. Do you think some people are more likely to respond positively to getting support to quit than others? Why? Could this be enhanced or improved? How well did any support you have had to quit address the barriers we discussed earlier? What could be done differently to assist you with the barriers to quitting for you? How might a relapse be prevented? Or what might be most helpful after relapse to get you back on the road to quitting?
- Do you have any other ideas about quitting smoking you would like to share?

At 8 months

- What has been your experience of attempts to Quit? Have you been successful? Have you relapsed? What do you think has been helpful or not helpful in your attempts to Quit? If you relapsed, why do you think this happened? What are the barriers you have faced in your attempts to quit – are there differences in the short term and the long term? What are the long-term barriers? How and why is it different? (prompts – environment, support people, smoking culture, mental health issues)
- What has been your experience of getting help to Quit? Have you had different needs over time depending on whether you have attempted to quit or not, or relapsed?
- Have you had assistance from peer workers? Do you think your attempts to quit were enhanced by involving peer workers? Why? How?
- Is there anything that could have been done differently to enhance the role of peer workers?

- What is your impression of the Quitline or other telephone counselling? (prompts – strengths, weaknesses, things you think could be done differently)
- What role did any telephone counselling you have had play in your attempts to Quit? Are there any other things that have been helpful (prompts – NRT, peer workers, engagement with mental health services and support network, peer worker follow up?). Please explain your thoughts on this. Do you think some people are more likely to respond positively to getting support to quit than others? Why? Does this change over time? Why? Could this be enhanced or improved? How well did any support you have had to quit address the barriers we discussed earlier? What could be done differently to assist you with the barriers to quitting for you? How might a relapse be prevented? Or what might be most helpful after a relapse to get you back on the road to quitting?
- Do you have any other ideas about quitting smoking you would like to share?
